# In Silico and RP HPLC Studies of Biologically Active 1,3,4-Thiadiazol-2-yl)-benzene-1,3-diols

**DOI:** 10.3390/molecules30193913

**Published:** 2025-09-28

**Authors:** Marek Studziński, Katarzyna Barańska, Beata Paw, Bogusław Senczyna, Tadeusz Paszko, Joanna Matysiak

**Affiliations:** 1 Department of Physical Chemistry, Faculty of Chemistry, Institute of Chemical Sciences, Maria Curie-Skłodowska University, 20-031 Lublin, Poland; marek.studzinski@umcs.pl; 2 Independent Radiopharmacy Unit, Medical University of Lublin, Chodzki 4a, 20-093 Lublin, Poland; k.baranska955@gmail.com; 3 Department of Medicinal Chemistry, Medical University of Lublin, Jaczewskiego St. 4, 20-090 Lublin, Poland; beata.paw@umlub.pl; 4 Department of Chemistry, University of Life Sciences in Lublin, Akademicka 15, 20-950 Lublin, Polandtadeusz.paszko@up.lublin.pl (T.P.)

**Keywords:** 1,3,4-thiadiazol-2-yl)-benzene-1,3-diol, HPLC, in silico, lipophilicity, IAM, log D_7.4_, RP C-18

## Abstract

Biologically active compounds from the 1,3,4-thiadiazol-2-yl)-benzene-1,3-diols group described earlier have been studied. Various approaches were used to determine their lipophilicity and predict pharmacokinetic properties. The lipophilicity parameters log k_w_ were determined using isocratic column chromatography and various stationary phases. Based on the standard curve and retention measurements by using an octadecyl column, the log D_7.4_ distribution coefficient was determined. A weak correlation was found between the experimentally determined log k_w_ parameters and the in silico calculated log P descriptors. It was shown that the compounds partially exist in an ionized anionic form at physiological pH. The determined log D_7.4_ parameter indicates that most of them have lipophilic character at the level recommended for potential drugs.

## 1. Introduction

Lipophilicity is one of the most important characteristics of bioactive compounds, whether potential drugs, pesticides, or poisons are taken under consideration. The lipophilic character is taken into account just at the stage of molecule design or at the earliest stage of biological research [1,2,3,4]. The lipophilicity parameter is included in the QSAR analysis; it is used to assess the pharmacokinetic and pharmacodynamic potential or to estimate the risks of using the substance and its possible impact on the natural environment [5,6,7,8,9,10].

Various chromatographic methods are commonly used to assess lipophilicity [11,12]. HPLC liquid column chromatography offers the greatest opportunities in this area [7,11,13,14]. It is also approved by OECD due to its reliability and simplicity [15].

The chromatographic methods are used in two possible ways [16]. The first one is based only on chromatographic retention data of a given series of compounds tested under the same conditions. Log k_w_ is the most commonly used parameter. This is a good solution when you need to compare the lipophilicity of a series of analogs to determine the effect of the various substituents on this parameter or when building QSAR models. Unfortunately, lipophilicity parameters obtained even in comparable chromatographic systems and conditions are not identical and do not correspond to the numerical values of the log P or log D determined using shake-flask extraction procedures. The obtained results depend on the type of organic modifier, the method of preparing the column with a specific packing, and many other not yet fully recognized variables. However, this approach is frequently used, and it is sufficient for the purpose of studies [17].

Another solution is to create and use a standard curve with known, extractively determined values of the partition (or distribution) coefficient [17,18]. This approach is based on the Collander equation [11,19]. It describes a linear correlation between log P values of neutral solutes and their log k_w_ parameters

log P = A log k_w_ + B (1)

where A and B represent constants of the linear regression analysis equation.

The octadecyl (C18) and octyl (C8) stationary phases with the water (or buffer)/organic modifiers (e.g., MeOH, ACN as the most popular) are officially recognized by both IUPAC and OECD as the chromatographic systems for experimental determination of lipophilicity of biologically active compounds [15,20,21,22]. However, more and more often the description of newly synthesized compounds is additionally replenished by the other systems with less common stationary phases in order to extend the amount of data describing possible biological behavior of investigated compounds in a more specific way [9,23].

Good examples of that type of chromatographic system are those based on application of immobilized artificial membrane (IAM). The silica surface of that phase was modified by immobilized phosphatidylcholine groups. IAM columns were elaborated and patented by Pidgeon et al. It is believed that retention on this stationary phase is the result of combination of hydrophobic, ion pairing, and hydrogen bonding interactions, and all of them are expected to be important in passive membrane transport, contrary to pure hydrophobic interactions revealing on alkyl bonded stationary phases [24,25,26]. Thus, the log k_w_ IAM values should be higher compared to log k_w_ RP-18 values if any interactions other than hydrophobic are present between the chromatographed compound and the stationary phase. IAM chromatography shows superior biomimetic properties to RP-18/8 since phosphatidylcholine is the major phospholipid present in cell membranes. Extrapolated log k_w_ parameters for the isocratic elution [17] and IAM chromatographic hydrophobicity indices (CHI/IAM) for gradient elution were used for the lipophilicity evaluation of compounds [5,11,27,28,29].

The second type of stationary phases which are used more often these days for the enhancement of chromatographic characterization of compounds are those with bonded cholesterol molecules. According to the investigation described by Buszewski et al., despite the marginally less hydrophobic character compared to C-18 stationary phases, they can mimic the cellular membrane very well and are very useful in prediction of permeability of xenobiotics across most of the biological membranes [30,31,32,33]. They are also believed to have good temperature robustness and stability without sacrificing the efficiency of separation and resolution [30,31].

Positively encouraged by the examples presented above, we decided to include the fifth type of stationary phase, the one with chemically bonded biphenyl groups present on the surface, in our investigation. According to earlier studies, the retention of the solutes on this type of stationary phase is contributed not only by hydrophobic interactions but also by π-π, steric, and hydrogen bond interactions. The combination of retention data obtained from all these columns should allow a detailed description of the investigated compounds [34,35,36].

The biologically active compounds from the 5-substituted 1,3,4-thiadiazol-2-yl)-benzene-1,3-diol group previously described have been studied [37,38,39]. They exhibit antiproliferative activity against the following human cancer cell lines: T47D (breast cancer), SW707 (rectal adenocarcinoma), and A549 (non-small cell lung carcinoma). The inhibitory effect was at the concentration of several µg/mL (ID_50_), and it was similar to that of cisplatin studied comparatively [37]. Some compounds show a prominent anticholinesterases effect. They are strong acetylcholinesterase (AChE) inhibitors with IC_50_ values of the order of magnitude of several nM for the most active derivatives and for moderate ones of butyrylcholinesterase (BuChE). Some derivatives exhibit high selectivity; the other ones are active against both enzymes [38]. 1,3,4-Thiadiazol-2-yl)-benzene-1,3-diols also have antifungal properties against the azole-resistant clinical isolates of *C. albicans* and nonalbicans *Candida* spp. [39]. Therefore, it is interesting to study them in various chromatographic systems, especially in those imitating biological systems.

The aim of the research is to analyze the phase affinity of biologically active compounds in terms of their ability to overcome the barrier in the form of biological membranes. Various stationary phases in HPLC chromatographic systems modeling biological systems were used for the lipophilicity determination. The log D_7.4_ parameter was also determined using a standard curve and C-18 HPLC measurements. Partition coefficients obtained by computational methods were also included in the investigation presented. Descriptive, correlation, and PCA analyses were used to study the dependencies among the obtained results for the investigated compounds.

## 2. Results and Discussion

### 2.1. Structure of the Studied Compounds

**Figure 1:**
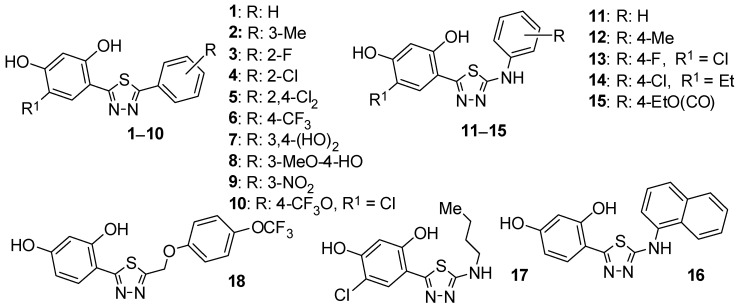
The molecular structures of the tested compounds (**1**–**18**).

The retention of biologically active compounds in the reversed-phase (RP) system was studied using different stationary phases and HPLC chromatography under isocratic conditions. Most of them are considered classic biomimetic systems. The 18 compounds from the 5-substituted 1,3,4-thiadiazol-2-yl)-benzene-1,3-diol group have been studied (Figure 1).

The structures were modified with aryl, alkyl/aryl, amino, and phenoxymethyl substituents. Additionally, some compounds contain a chlorine substituent in the polyphenol ring. On the one hand, the Cl atom increases the lipophilicity of the compound, and on the other hand, it increases the acidic character of the -OH groups of the resorcinol ring [37,38,39].

**Figure 2:**
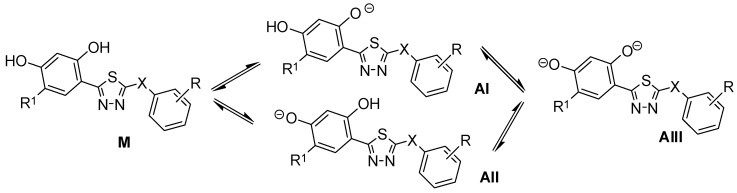
The dissociation process of 1,3,4-thiadiazol-2-yl)-benzene-1,3-diols.

The in silico calculations (Marvin, ver. 19.9) indicate that at pH 7.4, at which the chromatographic research was conducted, the compounds in the aqueous solution are generally in molecular or anionic form related to the dissociation of the ortho -OH group (Figure 2, Table 1).

The ratio of the molecular form is approximately 80–85% for most compounds, but in the case of the derivatives containing a chlorine atom in resorcinol moiety, they are much more dissociated (the molecular form is about 35%). This is the result of the electron-withdrawing effect of -Cl and the increase in the acidity of the -OH groups. Moreover, these compounds are characterized by higher acidity of the para -OH groups than the *ortho* ones (compounds **10**, **13**, **17**).

### 2.2. Log k_w_ Parameters

The retention factors of considered compounds were investigated using five different bonded stationary phases: octadecyl (RP-18), octyl (RP-8), cholesterol (Chol), phosphatidylcholine (IAM), and biphenyl (BPh). Water buffer–methanol mobile phases with pH 7.4 were used. The content of MeOH was varying and it depended on the type of compound and the stationary phase.

For the tested systems, linear relationships were obtained between the retention coefficient log k and the content of MeOH (%) in the mobile phase expressed by the Snyder–Soczewiński equation:

log k = log k_w_ + S (% organic modifier) (2)

where: log k_w_—the intercept; S—the slope of regression curve.

**Figure 3:**
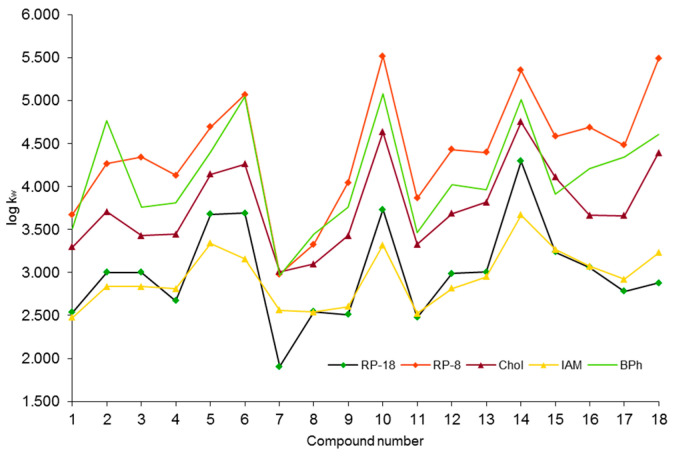
Log k_w_ values determined for investigated compounds based on experiments with RP-18, RP-8, Chol, IAM, and BPh columns.

Log k_w_ values were estimated by the extrapolation method. Log k_w_ and S parameters are presented in Figure 3, Table 2, and Tables S1–S5 in the Supplementary Materials. These parameters obtained by C-18, C-8, and IAM chromatography are commonly used as lipophilicity descriptors [16,40,41,42].

The comparison of investigated compound retention parameters (log k_w_) in different chromatographic systems is presented in Figure 3. The lowest log k_w_ values can be observed for RP-18 and IAM stationary phases. For most compounds, log k_w_ IAM values have similar values or are lower than those obtained for C18 stationary phase. This trend was observed for many other groups of compounds [43]. On the octadecyl column the main contribution to retention comes from almost entirely hydrophobic interactions, when in the case of IAM phase there are additional mechanisms involved in interactions as ion pairing and hydrogen bonding [24,25,26]. They can be either attractive or repulsive in nature. This depends on the structure of compound and pH. In some cases (where additional interactions contribute significantly to retention) this may change the order of the IAM and C18 log kw parameters. The highest log k_w_ values can be observed for RP-8 and BPh phases, which may suggest that the biggest retention contribution may come from residue silanol group spatial accessibility and interaction with an aromatic ring bonded with the stationary phase. Similar studies conducted with thiosemicarbazides using Chol, C-18, and IAM stationary phases showed the highest retention of compounds also on the cholesterol phase [17].

The weakest retention, thus, the lowest determined lipophilicity for all investigated stationary phases except IAM, can be observed for compound number **7** containing a double -OH groups on the second aromatic ring. Other investigated compounds with relatively low retention factor values are marked as **8**, **9,** and **11**. The strongest retained compounds are **6**, **10**, **14,** and **18** with the -OCF_3_ group or Cl atom substituted to the resorcinol ring.

The brief descriptive statistics regarding investigated group of compounds is presented in Table 2. The lowest mean absolute variability calculated as standard deviation of obtained values for tested compounds can be observed for the IAM phase (0.338), and the highest one for the octyl phase (0.695). Comparing it with standard C-18 variability, where pure hydrophobic interactions are present, one may say that residue silanol groups on a C-8 surface have a significant contribution to the retention. However, the highest relative variability for RP-18, and the lowest for IAM and cholesterol, may suggest that the presence of too many types of interactions can also have a negative influence on column selectivity and thus on the estimation of the influence of substituent presence on retention changes. C-8 and C-18 phases have the greatest range of absolute log k_w_ in the meaning of the greatest difference between the highest and lowest estimated values for investigated compounds, which allows a better recognition of lipophilicity changes of compounds and, in consequence, to quantify changes caused by differences among the analogues such as a constituent number and type or their location in relation to the unsubstituted basic structure.

The correlation coefficients R between the log k_w_ and S parameters for individual systems are about 0.9 (Table 3). These parameters are the best correlated for the C18 phase and the weakest for the BPh one.

### 2.3. Distribution Coefficient Log D_(7.4)_

Taking into consideration that dissociation degree of some investigated compounds is significantly different than others, which alters significantly their lipophilic character, log D_(7.4)_ was calculated to take that fact into consideration. C-18 chromatography was used to determine the distribution coefficient log D_(7.4)_ of the tested compounds using a standard curve. It was constructed for the reference compounds with known experimental log D_(7.4)_ values and studied chromatographically under the same conditions as the analyzed compounds [44] (Table 4). The obtained log k_w_ values were plotted against the log D_(7.4)_ based on BioLoom Database values taken from Andres et al. [44] (Table S6 in the Supplementary Materials). The following equation was obtained:

log D_(7.4)_ = −0.9184(±0.3996) + 1.3497(±0.1732) log k_w_ C18 (3)

n = 7, R = 0.9612, R^2^ = 0.9239, $R_{adj}^{2}$ = 0.9087, F(1,5) = 60.73, *p* < 0.00056, s = 0.4698

On the basis of Equation (3), log D_(7.4)_ values of all compounds were calculated (Table 4). The obtained log D_(7.4)_ values are very close to the log k_w_ values and the differences are less than 6% for most compounds. In the case of analogues **7** and **14**, these differences are the largest and they constitute about 17% and 13%, respectively (Table 4). This means that the obtained log k_w_ values from the C18/MeOH-water (pH = 7.4) chromatographic system reflect the extraction values of log D_(7.4)_ quite well. Some authors believe that log D was supposed to be taken into consideration in the “Rule of 5” instead of log P [45]. Yang et al. showed that the molecular feature of log D can help distinguish aggregators from non-aggregators in drug discovery [46].

### 2.4. Correlation Analysis

Obtained log k_w_ values from different stationary phases as lipophilicity parameters are compared in Table 5. They are also compared with those calculated using numerical methods—log P (Table S7 in the Supplementary Materials) [6,7]. The computational methods are very fast and allow calculation of this parameter for the designed compounds. Log k_w_ determined on BPh phase was also taken into account (Table 5).

The lipophilicity parameters obtained by different chromatographic systems are quite well-correlated with the partition coefficients log P calculated using the Marvin program (log P Axon, log P Cons). They are slightly less well-correlated with the S+log P and S+log D descriptors estimated by MedChem Designer. However, very weak correlations were found for Moriguchi log P (M logP), which is interchangeably used with Clog P in the “Rule of 5” [49,50].

Some applied in silico methods do not take into account phenomena such as solvation effect, tautomeric rearrangement, dissociation process, or hydrogen bond formation. Therefore, they may correlate poorly with lipophilicity parameters determined by chromatographic methods that take these processes into account. These phenomena may occur in the studied group of compounds and have a real impact on the lipophilicity.

Comparing the lipophilicity of the tested compounds in terms of recommendations for potential drugs, the obtained Mlog P values for all derivatives are within the recommended range (MlogP < 4.15) [50]. For these types of compounds, a high probability of favorable pharmacokinetic processes after oral administration is assumed. The optimal range of compounds lipophilicity expressed by log D_7.4_ covers the range 1–3. The data obtained indicate that most compounds have this parameter within the recommended range. Several of the most lipophilic analogues have this parameter in the range of around 3–5. It is assumed that these types of compounds are characterized by good permeability; however, the absorption is lower due to lower solubility in water [51].

Detailed data of the correlation analysis of selected chromatographic systems are presented in Equations (4)–(10). The results indicate that the cholesterol phase is the one that is the best correlated with other phases (Equations (4)–(6)):

log k_w_ C-8 = −0.4212(±0.4450) + 1.2806(±0.1170) log k_w_ Chol (4)

n = 18, R = 0.9392, R^2^ = 0.8821, $R_{adj}^{2}$ = 0.8747, F(1,16) = 119.72, *p* < 0.00000, s = 0.245

log k_w_ IAM = −0.5963(±0.2214) + 0.6218(±0.0582) log k_w_ Chol (5)

n = 18, R = 0. 9364, R^2^ = 0. 8769, $R_{adj}^{2}$ = 0. 8692, F(1,16) = 113.98, *p* < 0.00000, s = 0.1223

log k_w_ C-18 = −1.1530(±0.3531) + 1.1143(±0.0938) log k_w_ Chol (6)

n = 17, R = 0. 9507, R^2^ = 0.9039, $R_{adj}^{2}$ = 0. 8975, F(1,15) = 141.08, *p* < 0.00000, s = 0.1877

Compound **18** is an outlier.

A relatively high correlation is also found for the log k_w_ parameters obtained using the IAM and C18 stationary phases:

log k_w_ C18 = −1.6640 (±0.4845) + 1.5976 (±0.1646) log k_w_ IAM (7)

n = 17, R = 0.9287, R^2^ = 0.8625, $R_{adj}^{2}$ = 0. 8534, F(1,15) = 94.10, *p* < 0.00000, s = 0.2245

Compound **18** is an outlier. High correlations between these types of lipophilicity parameters are found for different groups of compounds [17,43].

Similar relations for lipophilicity parameters obtained by C-8 and C-18 chromatography were found (compound **18** is an outlier):

log k_w_ C18 = −0.5376(±0.3987) + 0.8162(±0.0908) log k_w_ C8 (8)

n = 17, R = 0.9183, R^2^ = 0.8434, $R_{adj}^{2}$ = 0.8329, F(1,15) = 80.75, *p* < 0.00000, s = 0.2397

The most similar log k_w_ values are found for the Chol and BPh phases (Equation (9)). This is evidenced by the slope close to 1 and the intercept of about 0.

log k_w_ BPh = −0.0944(±0.5962) + 1.0659(±0.1553) log k_w_ Chol (9)

n = 18, R = 0.8709, R^2^ = 0.7585, $R_{adj}^{2}$ = 0. 7424, F(1,15) = 47.11, *p* < 0.00001, s = 0.3080

This is also confirmed by the mean values from the descriptive statistics (Table 2).

The log k_w_ parameter obtained using the biphenyl phase is well correlated with that obtained from the C-8 phase (compound 2 is an outlier) Equation (10):

log k_w_ BPh = 0.5447(±0.3317) + 0.8003(±0.0742) log k_w_ C8 (10)

n = 17, R = 0.9411, R^2^ = 0.8856, $R_{adj}^{2}$ = = 08780, F(1,15) = 116.20, *p* < 0.00000, s = 0.2123

### 2.5. PCA Analysis

Principal component analysis is one of the methods of factorial analysis. Basing the analysis on the correlations (Pearson’s correlations in this study) allows the search for not only the correlations among pairs of chromatographic descriptors obtained from different columns but also allows for the comparison of all available data.

**Figure 4:**
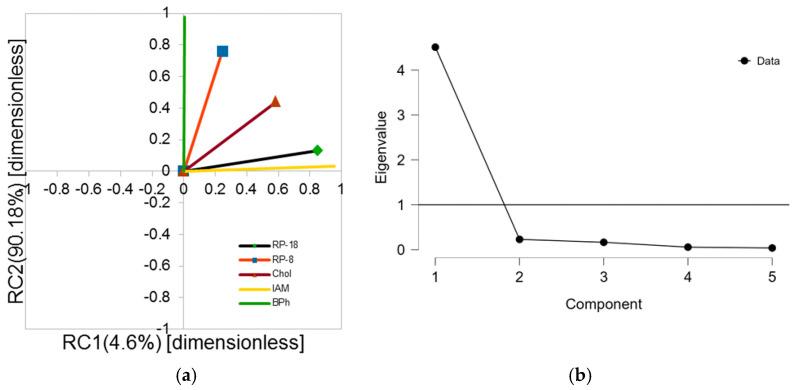
PCA loadings plot of chromatographic log k_w_ datasets for investigated compounds obtained for respective stationary phases (rotation: oblique, promax mode (**a**); PCA scree plot of chromatographic log k_w_ datasets for investigated compounds obtained for respective stationary phases (**b**) (detailed data can be found in Tables S8 and S9).

Similarly, as in the case of correlation analysis BPh stationary phase, which is also considered as the least similar to RP-18, this confirms the presence of probable π-π interaction retention contribution for the investigated compounds on the BPh column [34]. The most similar to RP-18 data seems to be IAM data, which confirms for those two columns that in their case the biggest contribution retention comes from hydrophobic interactions. Detailed results can be seen in Figure 4.

**Figure 5:**
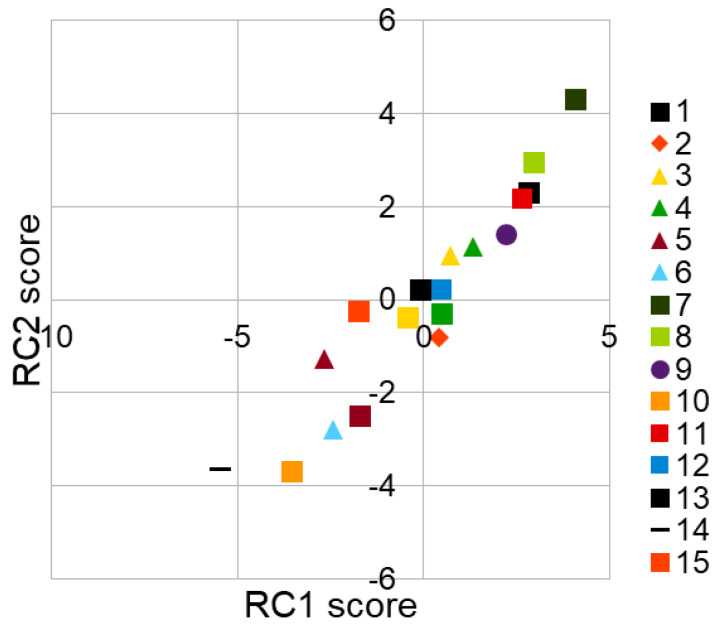
PCA score plot of chromatographic log k_w_ datasets for investigated substances obtained for respective stationary phases (rotation: oblique, promax mode).

The calculated relative contributions originating from log k_w_ values obtained from investigated systems for RC1 and RC2 present as follows: For RC1 component: RP-18—29.93%, IAM—3 7.93%, RP-8—8.89%, Chol—23.05%, BPh—0.2%. For RC2: RP-18—5.03%, IAM—1.37%, RP-8—30.53, Chol—19.43%, and BPh—43.64%. It confirms that first component is strongly correlated with hydrophobic interaction contribution to retention due to high IAM and RP-18 contributions. The high BPh value in the second component corresponds most probably to π-π interactions. Analyzing the score plot, which is the combination of results obtained in all investigated chromatographic systems in reference to respective studied compounds, may lead to many structure–chromatographic behavior related dependencies for investigated compounds (Figure 5). For example, the compounds with an unsubstituted external benzene ring (**1**, **11**) are very closely located to each other on the graph. The compounds with methyl group connect in a cluster near the center of the graph, which means that their scores are close to the mean value calculated for all compounds. The compounds with the -CF_3_ terminal group create a cluster in the lower left quarter of the graph in opposition to those containing nucleophilic substituents in locations 2 and 3 (3,4,9); they join in a cluster below on the left in relation to unsubstituted compounds, which allows for draw the conclusion that their character is similar to those containing the methyl group (or groups).

Compounds **7** and **8,** which contain the -OH group, are located up and right from unsubstituted **1** and **11;** thus, they are significantly different from those with methyl groups. In the central cluster, compounds **13** and **17** with Cl substituted to resorcyl ring can be also found close to the center of the graph. Despite the good connection between the position on the score plot PCA graph and the structure, not all the cases can be explained in this way (e.g., compounds **11** and **16**) due to yet unidentified reasons, which may need further investigation.

## 3. Materials and Methods

### 3.1. HPLC Measurements

HPLC measurements for C-18, C-8, IAM, Chol, and BPh phases were carried out using a liquid chromatograph Knauer (Knauer, Berlin, Germany) with a single pump working in isocratic mode, a 20 µL simple injection valve, and a UV–visible detector (Knauer, Berlin, Germany) working at 280 nm at room temperature. The retention time of an unretained solute (t_0_) was determined by the injection of a small amount of citric acid dissolved in water. The mobile phase flow rate was 1 mL/min (0.35 mL/min for Chol). HPLC columns: C18—Eurosil Bioselect 300 × 4 mm, 5 μm; C8—Waters. Symetry 150 × 4.6 mm, 5 μm, 100 Ǻ; IAM—Rexchrom IAM.PC.DD2 100 × 4.6 mm, 12 μm; Chol—Cogent 4 UDC Cholesterol 150 × 2.1 mm, 4 μm; BPh—Kinetex Biphenyl 150 × 4.6 mm, 5 μm were applied.

Mobile phase for RP-18, RP-8, Chol, and BPh chromatography was composed using 20 mM acetic buffer/MeOH with respective MeOH concentration ranges (volume fractions: C18—from 0.5 to 0.99; C8—from 0.5 to 0.9; IAM—from 0.15 to 0.5; Chol: from 0.45 to 0.9; BPh: from 0.4 to 0.99) to obtain pH = 7.4. In the case of IAM chromatography, phosphate buffer was used. At least five consecutive retention times for five concentrations per every investigated compound for every chromatographic system were measured. Log k parameter was calculated as log k = log (t_r_−t_0_/t_0_), where t_r_ = the retention time of the analyte; t_0_ = the retention time of an unretained compound.

### 3.2. Log D_(7.4)_ Determination from RP-18 Measurements

Ketoconazole, naphthalene, haloperidol, lidocaine, hydrocortisone, caffeine, and theophylline were used as references compounds. The analytical standards were purchased from Sigma Aldrich Steinhen ( MERCK, Darmstadt, Germany). The obtained log k_w_ values from HPLC C-18 were plotted against the log D_(7.4)_ based on BioLoom Database values taken from Andres et al. [44] (Table S6 in the Supplementary Materials).

### 3.3. Calculation Methods

The estimation of pK values, the percentage of the compounds, individual forms calculations, and log P Axon and log P Cons calculations were made by Marvin ver. 19.9 [47]. The calculations of S+log D, S+log P, and Mlog P were made by the MedChem Designer ver. 5.5.0.11 [48]. Statistica ver. 7.1 was used for the regression and correlation analysis [52] and JASP ver. 0.17.3 for PCA analysis [53].

## 4. Conclusions

The studies conducted indicate that the investigated compounds exist in both molecular and anionic forms at physiological pH equal to 7.4. This is an expected feature of potential drugs, enabling both solubility in aqueous media and the crossing of barriers such as biological membranes. The degree of dissociation depends on pH, and it can be assumed that such compounds may be well absorbed in the stomach.

Weak correlations were obtained between the experimentally determined log k_w_ parameters and the in silico-determined log P descriptors. This indicates the need to use chromatographic methods for a detailed assessment of the lipophilicity of the thiadiazoles studied and all newly synthesized compounds in general.

Studies have also shown that computational methods are not the best solution for estimating lipophilicity for pharmacokinetic prediction purposes in the cases of these types of compounds. In some cases, they provide a different classification of the compound than experimental measurements. This applies to the most lipophilic compounds. A better solution is to undertake chromatographic measurements using a standard curve, which can be related to the log D_7.4_ scale. Importantly, this procedure is neither tedious nor time-consuming. Computational methods are certainly a very good solution for determining the log P parameter of compounds currently in the design phase.

## Figures and Tables

**Table 1:** The percentage of molecular (M) and anionic forms (AI–AIII) of compounds at pH 7.4.

Compound No.	Molecular form (M) [%]	Anion AI [%]	Anion AII [%]	Anion AIII [%]
**1**	81.95	14.55	3.36	0.13
**2**	81.95	14.56	3.37	0.13
**3**	81.4	14.99	3.47	0.14
**4**	81.65	14.79	3.42	0.13
**5**	81.65	14.79	3.42	0.13
**6**	81.95	14.56	3.36	0.13
**7**	77.08	13.69	9.20 ^1^	-
**8**	80.44	14.29	4.27 ^1^	-
**9**	60.73	34.27	4.46	0.55
**10**	32.55	46.26 ^2^	15.92 ^3^	5.27
**11**	84.27	12.71	2.92	0.1
**12**	84.27	12.71	2.92	0.1
**13**	36.54	44.06 ^2^	15.13 ^3^	4.26
**14**	92.38	5.79	1.8	0.03
**15**	84.27	12.71	2.92	0.1
**16**	84.26	12.72	2.92	0.1
**17**	37.44	43.55 ^2^	14.95 ^3^	4.06
**18**	79.98	16.12	3.74	0.16

^1^ Other anionic forms related to the dissociation of other -OH groups. ^2^ The percentage of anionic form AII. ^3^ The percentage of anionic form AI.

**Table 2:** The comparison of retention (log k_w_) of investigated compounds and descriptive statistics in various chromatographic systems.

Parameter	RP-18	IAM	RP-8	Chol	BPh
Mean	3	2.94	4.406	3.77	4.116
Std. Deviation	0.57	0.338	0.695	0.509	0.612
RSD [%]	19	11.497	15.774	13.501	14.869
Minimum	1.907	2.478	2.98	3.01	2.965
Maximum	4.296	3.67	5.517	4.751	5.075

**Table 3:** The correlation coefficient r between the log k_w_ and S parameter for individual chromatographic systems.

System	C-18	IAM	C-8	Chol	BPh
R	−0.968	0.905	0.937	0.908	−0.885

**Table 4:** Chromatographic parameters of compounds obtained by C18 stationary phase (log k_w_, S, R^2^) and log D_(7.4)_ calculated from standard curve.

No.	log k_w_ C-18	S	R^2^	log D_(7.4)_	Δ ^1^	E [%] ^2^
**1.**	2.537	−3.494	0.981	2.506	−0.032	−1.267
**2.**	3	−3.87	0.987	3.131	0.157	4.970
**3.**	3.002	−3.885	0.983	3.133	0.158	4.991
**4.**	2.67	−3.651	0.990	2.685	0.022	0.833
**5.**	3.678	−4.438	0.993	4.046	0.433	10.535
**6.**	3.69	−4.696	0.997	4.062	0.438	10.611
**7.**	1.907	−3.171	0.941	1.656	−0.288	−17.820
**8.**	2.545	−3.785	0.98	2.517	−0.028	−1.132
**9.**	2.512	−3.432	0.974	2.472	−0.042	−1.698
**10.**	3.734	−4.65	0.98	4.121	0.456	10.882
**11.**	2.477	−3.66	0.977	2.425	−0.056	−2.321
**12.**	2.986	−4.168	0.99	3.112	0.151	4.819
**13.**	3.005	−4.173	0.997	3.138	0.159	5.023
**14.**	4.296	−5.335	0.997	4.880	0.685	13.751
**15.**	3.238	−4.185	0.975	3.452	0.254	7.270
**16.**	3.059	−3.931	0.987	3.210	0.181	5.584
**17**	2.78	−3.931	0.987	2.834	0.067	2.362
**18.**	2.879	−3.883	0.948	2.967	0.108	3.602

^1^ The differences calculated as Δ = log D_(7.4)_ − log k_wRP-18_. ^2^ Relative error calculated as Δ/log D_7.4_ × 100%.

**Table 5:** Correlation matrix (r) of log k_w_ values obtained by HPLC at various stationary phases and log P parameters estimated by various computational methods.

Descriptor	log k_w_ C-18	log k_w_ IAM	log k_w_ C-8	log k_w_ Chol	log k_w_ BPh
log k_w_ C-18	1	-	-	-	-
log k_w_ C-8	0.918 (18) ^3^	-	1	-	
log k_w_ IAM	0.929 (8) ^3^	1	0.871	-	
log k_w_ Chol	0.920 (18) ^3^	0.938	0.94	1	-
log k_w_ BPh	0.844	0.797	0.943 (2) ^3^	0.867 (2,6) ^3^	1
log P Axon ^1^	0.848 (8) ^3^	0.866 (10) ^3^	0.914	0.859 (2,6)	0.867 (2,6) ^3^
log P Cons ^1^	0.883 (18) ^3^	0.855 (10) ^3^	0.9	0.909	0.859 (2,6) ^3^
Mlog P ^2^	0.65	0.435	0.555	0.435	0.625
S+log P ^2^	0.788	0.663	0.719	0.817 (15,18) ^3^	0.772
S+log D ^2^	0.841 (4) ^3^	0.699	0.848 (5,18) ^3^	0.699	0.761

^1^ Log P models calculated by Marvin, version 19.9 software [47]. ^2^ Log P calculated by MedChem Designer software [48]. ^3^ Outlier compounds.

## Data Availability

All data is available in the ‘Supplementary Materials’ of this contribution.

## Supplementary Materials

The following supporting information can be downloaded at: https://www.mdpi.com/article/10.3390/molecules30193913/s1: Table S1: Log k_w_ and S values obtained for C-18/methanol–water chromatographic system; Table S2: Log k_w_ and S values obtained for RP-8/methanol–water chromatographic system; Table S3: Log k_w_ and S values obtained for IAM//methanol–water chromatographic system; Table S4: Log k_w_ and S values obtained for Chol/methanol–water chromatographic system; Table S5: Log k_w_ and S values obtained for BPh/methanol–water chromatographic system; Table S6: Log k_w_ C-18 values and log D_(7.4)_ of standards used for calibration curve construction; Table S7: Values of lipophilicity descriptors calculated with Marvin ver. 19.9 (log P Axon, log P Cons) and MedChem Designer ver. 5.5.0.11 (Mlog P, S+log P, S+log P) software [47,48] for investigated compounds; Table S8: Component loadings for PCA analysis of data obtained from retention measurements on different columns; Table S9: Component characteristics for PCA analysis of data obtained from retention measurements on different columns.
