# Supplementary material for: In Silico and RP HPLC Studies of Biologically Active 1,3,4-Thiadiazol-2-yl)-benzene-1,3-diols
Source: Molecules. 2025 Sep 28;30(19):3913. doi: 10.3390/molecules30193913 (PMC12525849; doi:10.3390/molecules30193913)
Supplement: Supplementary file 1 [file molecules-30-03913-s001.zip › molecules-3875508-supplementary.pdf]

In silico and RP HPLC studies of biologically active 1,3,4-thiadiazol-2-yl)-benzene-1,3-diols

Marek Studziński, Katarzyna Barańska, Beata Paw, Bogusław Senczyna , Tadeusz Paszko, and Joanna Matysiak

Marek Studziński [1], Katarzyna Barańska[2], Beata Paw [3], Bogusław Senczyna [4], Tadeusz Paszko [4], and Joanna Matysiak [4]

[1] Maria Curie-Skłodowska University, Institute of Chemical Sciences, Faculty of Chemistry, Department of Physical Chemistry, Lublin, Poland.

[2] Independent Radiopharmacy Unit, Medical University of Lublin, Chodzki 4a, PL-20093 Lublin, Poland.

[3] Medical University of Lublin, Department of Medicinal Chemistry, Jaczewskiego St. 4, 20-090 Lublin, Po-land.

[4] University of Life Sciences in Lublin, Department of Chemistry , Akademicka 15, 20-950 Lublin, Poland.

Table S1. Log k_w and S values obtained for C-18/methanol-water chromatographic system.

Compound No.	R^2	log k_w	S
**1**	0.981	2.537	-3.494
**2**	0.987	3.000	-3.870
**3**	0.983	3.002	-3.885
**4**	0.990	2.670	-3.651
**5**	0.993	3.678	-4.438
**6**	0.997	3.690	-4.696
**7**	0.941	1.907	-3.171
**8**	0.980	2.545	-3.785
**9**	0.974	2.512	-3.432
**10**	0.980	3.734	-4.650
**11**	0.977	2.477	-3.660
**12**	0.990	2.986	-4.168
**13**	0.997	3.005	-4.173
**14**	0.997	4.296	-5.335
**15**	0.975	3.238	-4.185
**16**	0.987	3.059	-3.931
**17**	0.987	2.780	-3.931
**18**	0.948	2.879	-3.883

Table S2. log k_w and S values obtained for RP-8/methanol-water chromatographic system.

Compound No.	R^2	log k_w	S
**1**	0.993	3.674	-5.105
**2**	0.994	4.266	-5.863
**3**	0.982	4.341	-5.468
**4**	0.993	4.130	-5.579
**5**	0.992	4.691	-5.807
**6**	0.990	5.070	-7.056
**7**	0.996	2.980	-5.165
**8**	0.984	3.325	-4.835
**9**	0.984	4.045	-6.986
**10**	0.991	5.517	-6.989
**11**	0.989	3.862	-6.800
**12**	0.986	4.428	-6.966
**13**	0.987	4.395	-6.032
**14**	0.994	5.351	-7.042
**15**	0.938	4.580	-6.264
**16**	0.987	4.687	-5.919
**17**	0.995	4.480	-5.897
**18**	0.989	5.489	-7.743

Table S3. log k_w and S values obtained for IAM//methanol-water chromatographic system.

Compound No.	R^2	log k_w	S
**1**	0.990	2.478	-5.22
**2**	0.989	2.837	-5.74
**3**	0.989	2.837	-5.75
**4**	0.988	2.812	-5.64
**5**	0.987	3.337	-6.34
**6**	0.993	3.156	-6.23
**7**	0.979	2.562	-5.81
**8**	0.980	2.539	-5.75
**9**	0.990	2.603	-5.4
**10**	0.994	3.318	-6.19
**11**	0.988	2.525	-5.33
**12**	0.990	2.813	-5.79
**13**	0.996	2.949	-5.78
**14**	0.997	3.670	-6.689
**15**	0.987	3.269	-6.48
**16**	0.989	3.074	-6.18
**17**	0.993	2.920	-6.07
**18**	0.995	3.227	-6.69

Table S4. Log k_w and S values obtained for Chol//methanol-water chromatographic system.

Compound No.	R^2	log k_w	S
1	0.984	3.296	-3.916
2	0.996	3.708	-4.460
3	0.998	3.427	-4.161
4	0.997	3.446	-4.257
5	0.998	4.144	-4.657
6	0.998	4.259	-5.105
7	0.994	3.010	-4.333
8	0.984	3.098	-4.108
9	0.999	3.427	-4.181
10	0.999	4.632	-5.519
11	0.991	3.328	-4.300
12	0.999	3.686	-4.601
13	0.998	3.816	-4.628
14	0.997	4.751	-5.322
15	0.998	4.109	-4.907
16	0.997	3.664	-4.531
17	0.998	3.661	-4.777
18	0.999	4.391	-5.665

Table S5. Log k_w and S values obtained for BPh//methanol-water chromatographic system.

Compound No.	R^2	log k_w	S
1	0.997	3.512	-4.250
2	0.986	4.765	-5.863
3	0.969	3.761	-4.586
4	0.998	3.814	-4.517
5	0.999	4.404	-5.077
6	0.975	5.041	-6.447
7	0.996	2.965	-4.670
8	0.991	3.450	-4.869
9	0.999	3.756	-4.416
10	0.998	5.075	-6.415
11	0.998	3.465	-4.639
12	0.996	4.024	-5.233
13	0.998	3.965	-5.298
14	0.996	5.011	-6.165
15	0.999	3.910	-4.965
16	0.997	4.205	-5.121
17	0.995	4.346	-4.928
18	0.998	4.609	-5.857

Table S6. Log k_w C-18 values and **log $D_{(7.4)}$** of standards used for calibration curve construction.

Standard Name	R^2	log k_w C-18	log $D_{(7.4)}$ Bio Loom
ketoconazole	0.977	3.370	3.83
naphthalene	0.998	2.615	3.3
haloperidol	0.996	3.012	2.92
lidocaine	0.997	2.079	1.53
hydrocortisone	0.998	2.210	1.61
caffeine	0.977	0.802	-0.07
theophylline	0.982	0.380	-0.02

Table S7. Values of lipophilicity descriptors calculated with Marvin ver. 19.9 [1] (log P Axon , log P Cons) and MedChem Designer ver. 5.5.0.11 (Mlog P, S+log P, S+log P) software [2] for investigated compounds.

Compound No.	log P Axon	logP Cons	Mlogl P	S+log P	S+log D
1	2.88	3.19	2.716	3.534	3.49
2	3.35	3.7	2.972	3.864	3.828
3	3.02	3.33	2.845	3.603	3.540
4	3.4	3.79	2.972	3.961	3.855
5	3.92	4.4	3.221	4.542	4.373
6	3.76	4.07	3.344	4.323	4.276
7	2.31	2.58	1.156	2.394	2.335
8	2.34	2.73	1.412	2.580	2.523
9	2.84	3.13	2.495	3.167	3.055
10	5.08	5.23	2.796	4.435	4.069
11	2.85	3.29	2.334	3.027	2.994
12	3.31	3.8	2.590	3.402	3.375
13	3.5	4.04	2.716	4.063	3.718
14	4.23	4.85	3.083	4.151	4.120
15	3.34	3.85	1.882	3.215	3.191
16	3.85	4.28	3.098	4.020	3.995
17	2.89	3.25	2.310	3.697	3.468
18	4.16	4.24	2.024	3.312	3.266

[1] Marvin, version 19.9, ChemAxon Ltd, 2024.
[2] MedChem Designer(TM), version 5.5.0.11; Simulations Plus, Inc.

Table S8. Component loadings for PCA analysis of data obtained from retention measurements on different columns.

Phase	RC1	RC2	Uniqueness
IAM	0.957	0.031	0.035
RP-18	0.850	0.128	0.083
Chol	0.584	0.441	0.041
RP-8	0.246	0.757	0.060
BPh	0.005	0.975	0.042

Table S9. Component characteristics for PCA analysis of data obtained from retention measurements on different columns.

	Unrotated solution			Rotated solution		
	Eigenvalue	Proportion var.	Cumulative	SumSq. Loadings	Proportion var.	Cumulative
Component 1	4.509	0.902	0.902	1.148	0.230	0.230
Component 2	0.230	0.046	0.948	1.247	0.249	0.479
Component 3	0.165	0.033	0.981	1.069	0.214	0.693
Component 4	0.057	0.011	0.992	1.097	0.219	0.912
Component 5	0.039	0.008	1.000	0.439	0.088	1.000
